# Glucose indices as inflammatory markers in children with acute surgical abdomen: a cross-sectional study[Author-notes FN0001]

**DOI:** 10.1080/07853890.2023.2248454

**Published:** 2023-10-20

**Authors:** Hoda Atef Abdelsattar Ibrahim, Sherif Kaddah, Sara Mohamed Elkhateeb, Abeer Aboalazayem, Aya Ahmed Amin, Mahmoud Marei Marei

**Affiliations:** a Paediatric Clinical Nutrition Unit, Paediatric Department, Faculty of Medicine, Cairo University, Cairo, Egypt; b Paediatric Surgery Section/Unit, Cairo University Hospitals (Cairo University Specialized Paediatric Hospital [CUSPH] & Cairo University Children’s Hospital [Abu El-Reesh El-Mounira]), Department of Surgery, Faculty of Medicine, Cairo University, Cairo, Egypt; c Clinical Pathology Department, Faculty of Medicine, Cairo University, Cairo, Egypt; d Cancer Epidemiology and Biostatistics, National Cancer Institute, Cairo University, Cairo, Egypt

**Keywords:** Glycaemic Dysregulation, Glycated Haemoglobin (HbA1c), Random Blood Sugar (RBS), Stress Hyperglycaemia, Inflammatory Markers, Acute Abdomen, Acute Appendicitis, Paediatric Surgical Emergencies

## Abstract

**Background:**

Glycaemic dysregulation potentiates the pro-inflammatory response and increases oxidative injury; therefore, preoperative hyperglycaemia is linked to increased mortalities. In addition, inflammation is accompanied by higher glycated haemoglobin (HbA1c) levels, and the relationship between this and random blood sugar (RBS) could be non-linear.

**Methods:**

This is a cross-sectional study. Non-diabetic paediatric patients with acute surgical abdomen, presenting to the emergency surgical services were enrolled, over a period of 6 months. They were all screened for their random blood sugar and HbA1c levels.

**Results:**

Fifty-three cases were studied. The prevalence of glycaemic dysregulation in the enrolled children was high. Abnormal HbA1c was observed in 66% of the study group. Stress hyperglycaemia was observed in 60% of the enrolled children. There was a significant correlation (*r* = 0.770, *p*-value: < 0.001) between RBS and the total leucocytic count (TLC). The TLC cutoff value for predicting stress hyperglycaemia was 13,595 cells/mm^3^. The cutoff value of RBS for predicting leukocytosis was 111.5 mg/dl. Median RBS level was significantly higher in complicated appendicitis (169.5 mg/dl), compared to uncomplicated appendicitis (118.0 mg/dl).

**Conclusion:**

HbA1c and RBS could be used as inflammatory markers for surgical acute abdomen and its degree of severity, respectively. HbA1c rises in a considerable number of cases with surgical acute abdomen, irrespective of the disease stage. However, as the disease progresses, the random blood sugar rises due to stress hyperglycaemia, thus becoming a surrogate inflammatory marker.

## KEY MESSAGES

1. **What is already known on this topic?** Preoperative stress hyperglycaemia is common in children, and it is linked to adverse postoperative outcomes. HbA1c could be a marker for inflammation and oxidative stress.
2. **What does this study add?** HbA1c could be an inflammatory marker for surgical acute abdomen, irrespective of the disease stage, as it had a high prevalence in the enrolled children with an acute surgical abdomen. However, as the disease progresses, the random blood sugar rises due to stress hyperglycaemia, thus becoming a surrogate inflammatory marker, as there is a significant correlation between it and the TLC.
3. **How might this study affect research, practice, or policy?** The novelty in this study centers around the role of glucose metabolism, as evaluated by random blood sugar and HBA1c, in the diagnostic evaluation and prognostication of inflammation, represented by the surgical acute abdomen. This may invite further research into understanding the underlining mechanisms. The outcome of the clinical management of conditions involving inflammation can be improved by using the proposed biomarkers, as peri/preoperative hyperglycaemia could lead to morbidity and mortality, consequently, as proven, the reliability of those biomarkers facilitates risk assessment and stratification. As both tests are cost-effective and universally available, they can be readily implemented in practice guidelines and departmental policies.

## 1. Introduction

During surgical procedures, blood glucose level rises as a normal physiological response to tissue damage, potential sepsis, and surgical trauma, which is known as stress hyperglycaemia, and occurs regardless of the diabetic status. Moreover, diabetic patients are subjected to a higher risk of hyperglycaemia due to several added pathophysiologic changes, such as impairment of the vascular endothelium and cell-level damage, leading to cardiovascular dysfunction. This aggravates morbidity and may lead to mortalities [1,2].

Numerous cellular and biochemical events set the scene for the development of adverse outcomes related to and/or in response to hyperglycaemia. For instance, the risk of infection is increased with severe hyperglycaemia, owing to abnormalities of monocyte and polymorphonuclear neutrophil function, decreased intracellular bactericidal activity, and the glycosylation of immunoglobulins [3]. Besides, blood coagulation is enhanced by hyperglycaemia, as circulating prothrombin fragments and D-dimers are amplified, and platelet aggregation and thrombosis may ensue, leading to a hypercoagulable state [4].

HbA1c reflects the average plasma glucose over the preceding 8 to 12 weeks. It can be measured at any time and does not need fasting. These properties have made it the chosen test for assessing glycaemic control in patients with diabetes mellitus (DM) [5]. HbA1c could have more uses than the evaluation of glycaemic control. Higher levels of it are associated with hypercoagulability and inflammation, but whether this feature is independent of blood glucose concentration remains uncertain. It was observed that HbA1c reflects the effects of inflammation in nondiabetic individuals and may be a marker of inflammation risk, independent of fasting blood glucose, race, and obesity [6]. Additionally, some recent reports suggest that HbA1c may have prognostic values in patients with acute ischemia [7]. A former study found that incremental increases in HbA1c levels are linked to higher hazards of adverse limb outcomes, independent of preoperative DM status. Poor glycaemic control (HbA1c >7.0%) in patients without a preoperative DM diagnosis conveys twice the relative risk of amputation than in those with good glycaemic control. Several studies evaluated the prognostic value of HbA1c in nondiabetic patients. It was detected that the elevated HbA1c level is associated with mortalities [8]. This is of particular importance in the context of the current study, as there is an ischaemic component in the pathogenesis of acute surgical abdomen.

To the best of our knowledge, few studies investigated the glycaemic status in non-diabetic children in the setting of the acute surgical abdomen. Our study was carried out to outline the possible usability of the glycaemic status for risk stratification and whether HbA1c could be used as an inflammatory marker in children with acute abdomen.

## 2. Patients/materials and methods

### 2.1. The current study design

This is a cross-sectional analytical study.

### 2.2. Study area and setting

The current study was carried out at the paediatric surgical department of Cairo University Children’s Hospitals, for all children admitted with the acute surgical abdomen, over a period of 6 months, from 1^st^ December 2021 to the end of May 2022.

### 2.3. Criteria of the enrolled children in the study

1. ***Inclusion criteria****:* (A) Paediatric patients (beyond 1 month old up to 12 years old.) (B) Patients with acute abdomen presenting to the pediatric surgical emergency services, before receiving intravenous fluids or glucose that could affect their RBS. (C) Patients with no past history suggestive of diabetes mellitus or any treatment for it. (D) Patients whose parents or caregivers agreed for them to be enrolled in the study.
2. ***Exclusion criteria***: Paediatric patients who presented to the paediatric (medical or surgical) emergency services with (A) diagnoses other than acute abdomen, (B) were diabetic children; and (C) patients whose parents or caregivers did not agree to be enrolled in the study were excluded.

### 2.4. Sampling technique for the enrolled children

A consecutive sample of all children with acute abdomen (*n* = 53) who were admitted to the emergency department, with acute abdomen, and managed by the paediatric surgical department of Cairo University Children’s Hospitals, were recruited for the current study, throughout the study period.

### 2.5. Data collection from the enrolled children

1. Demographic and clinical criteria:
  - Age and gender
  - Causes of acute abdomen (primary diagnosis)
  - Clinical picture suggestive of inflammation of the enrolled children, including fever and chills (rigours)
2. Laboratory criteria including Full/Complete blood count (FBC or CBC), random blood sugar (RBS) and HbA1c:
  - HbA1c: HbA1c was measured using Tosoh G8 HPLC Analyzer, a fully automated A1C analyzer that utilizes the Ion-Exchange High-Performance Liquid chromatography method (TOSOH G8, South San Francisco, CA).
  - RBS: reducing methods.
  - CBC (FBC): Automated cell counter Beckman Coulter DxH 690T.

### 2.6. Outcomes measured

- The primary objective of our study was to assess the prevalence of stress hyperglycaemia in paediatric patients with a surgical acute abdomen.
- Secondary outcomes included the relation between the glucose indices in the form of RBS and HbA1c, and the inflammatory laboratory markers as total leucocytic count, and detection of any corelation between RBS and HbA1c.

### 2.7. Operational definitions

1. Hyperglycaemia, using reference ranges of RBS. Its normal range is from 65 mg/dl to 99 mg/dl [9]
2. Leukocytosis, using reference ranges of TLC. This may vary according to the age e.g. in children aged from 1-2 years, its normal range is from 6000 mm^3^ to 17,000 mm^3^; and in those aged from 2-4 years, its normal range is from 5500 mm^3^ to 15,500 mm^3^ [9].
3. Levels of HBA1c, using the reference ranges previously reported [10].
4. Acute abdomen: The first step in the diagnostic pathway was clinical evaluation. A preliminary diagnosis was made based on medical history, physical examination, and, in enrolled cases, laboratory parameters. Symptoms and signs of acute abdomen included but were not restricted to abdominal pain, whether localised or diffuse, abdominal tenderness, tense (rigid) abdominal muscles on palpation denoting guarding, distended abdomen and/or rapid shallow breathing [3].
5. Complicated appendicitis is defined as perforated appendicitis, peri-appendicular abscess, or peritonitis [11].

### 2.8. Sample size calculation

The primary objective of the study was to assess stress hyperglycaemia in hospitalized children with surgical acute abdomen. Russo et al. (2017) reported that stress hyperglycaemia was detected in 12.13% of hospitalized patients [12]. Epi Info (version 7.2.) software was used for sample size estimation. By setting alpha as 0.05, a confidence level of 95% and an acceptable margin of error of 0.10, the minimum required sample size for the study was calculated to be 41 patients.

### 2.9. Ethical considerations

The study was revised and approved by the Research Ethics Committee of Cairo University, Faculty of Medicine**,** ethical clearance number N-97-2021. The study was completed in accordance with the legislations, regulations and directives for human-related medical research. Informed consent was obtained from the parents or caregivers of the enrolled children. This study followed the principles of the World Medical Association (WMA) Declaration of Helsinki and Good Clinical Practice (GCP) guidelines and framework. Consent for any clinically indicated medical management or surgical procedure/intervention followed the standard informed and written documentation.

### 2.10. Statistical methods

Statistical calculations were conducted using SPSS (Statistical Package for the Social Science; SPSS Inc., Chicago, IL, USA) version 24. Categorical variables were described as frequency counts and percentages, while numerical variables were described as mean and standard deviation or median and range, as appropriate. Association between categorical variables were tested using the chi-square test or Fisher exact if the expected cell count was less than 5 in more than 25% of cells. For comparing medians of non-normally distributed variables, the Kruskal Wallis test was used, followed by pairwise comparisons with Bonferroni adjustment. Kendall’s Tau b correlation was used to test the correlation between numeric non-normally distributed variables. Alpha was set at 0.05. The receiver operator (operating) characteristic curve (ROC) calculations were used in detecting the cutoff value of stress hyperglycaemia for predicting TLC (and vice versa), and for each (RBS and TLC) to predict complicated appendicitis. Cutoff values with the highest balanced levels of sensitivity and specificity were chosen. Predictive values of positive and negative tests were calculated.

## 3. Results

**Figure 1.**
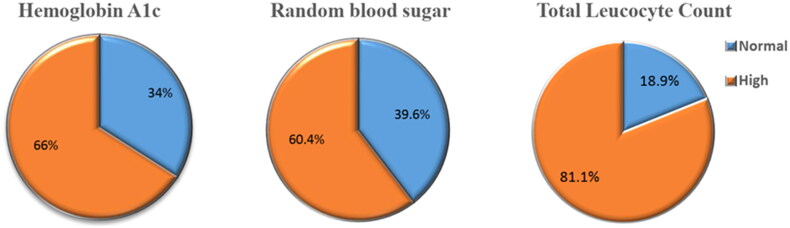
The prevalence of abnormal Haemoglobin A1c, random blood sugar and total leucocyte count, among the studied group of patients.

The enrolled children showed a male predominance (66%). The mean age of the study participants was 7.8 years (Table 1). Acute appendicitis was the primary diagnosis in 84.9% (*n* = 45) of the studied group of patients, 58.5% (*n* = 31) had uncomplicated appendicitis, while 26.4% (*n* = 14) were complicated appendicitis. The prevalence of stress hyperglycaemia was high among children with acute abdomen, as it was observed in 60.4% (*n* = 32) of the study participants. Besides, abnormal HBA1c existed in a considerable number of cases (66%, *n* = 35). Most enrolled children (81%) were found to have leukocytosis (Figure 1).

**Table 1.** Demographic criteria of the study participants.

Demographic characteristics		
Age (years)		
Mean ± SD*	7.8 ± 3.8	
Median (Range)	9.0 (0.2 − 12.0)	
Gender	Number (*n*)	Percentage (%)
Male	35	66.0
Female	18	34.0
Total	53	100.0

* SD: Standard Deviation.

The TLC was significantly different among patients of different diagnoses (*p*-value: 0.001) (Table 2). By performing posthoc pairwise comparisons with Bonferroni adjustments, the median TLC count in patients diagnosed with uncomplicated appendicitis (13,400 cell/mm^3^) was significantly different from that of patients with complicated appendicitis (28,050 cell/mm^3^) with a *p*-value of 0.002 (Table 2).

**Table 2.** Total leucocyte count and RBS among different diagnoses of the study participants.

	Uncomplicated Acute Appendicitis (n = 31)	Complicated Appendicitis (n = 14)	Intussusception (n = 7)	*P* value
Total leucocyte count (cell/mm^3^)				
Median	13,400	28050	13,490	0.001
Interquartile range	10,700–20,900	19,950 − 30,250	10,300–14,650	
Random blood sugar (mg/dl)				
Median	118.0	169.5	127.0	0.001
Interquartile range	(97.0 − 146.0)	(134.5 − 179.2)	(90.0 − 136.0)	

* Irreducible hernia could not be included in this analysis because there was only one case.

**Figure 2.**
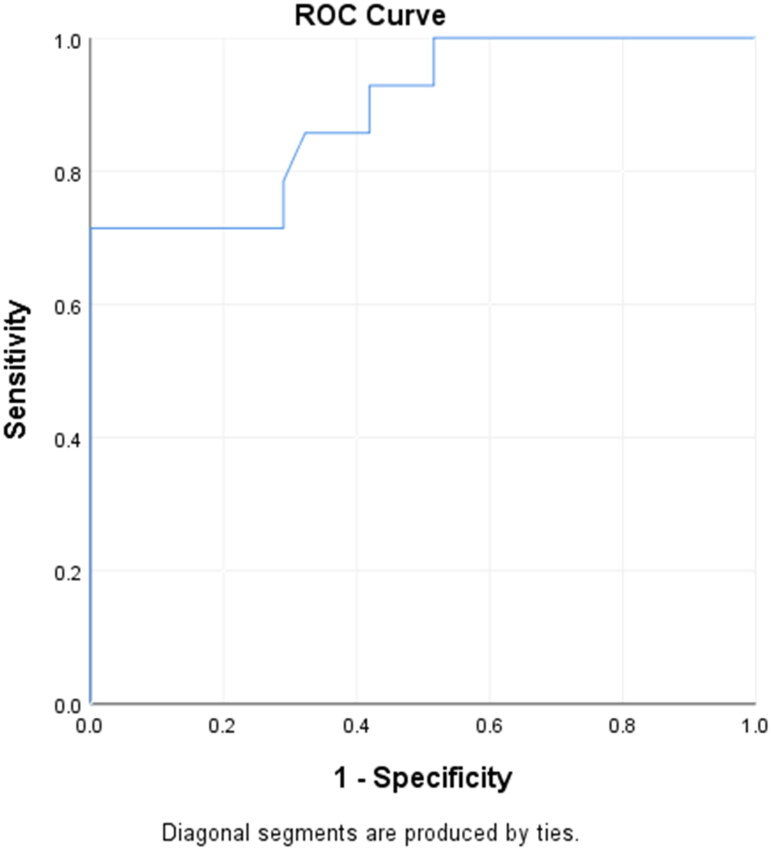
Receiver operator characteristic curve (ROC) for total leukocyte count in detecting complicated appendicitis.

By performing the Receiver Operating/Operator Characteristic (ROC) curve analysis, the TLC cutoff value for predicting complicated appendicitis was 18,650 cells/mm^3^. The sensitivity was 85.7%, specificity was 67.7%, positive predictive value (PPV) was 54.5%, and negative predictive value (NPV) was 91.3%, at this TLC cutoff value (18,650 cells/mm^3^). The Area Under the Curve (AUC) was 0.891, as represented by the ROC curve (Figure 2).

**Figure 3.**
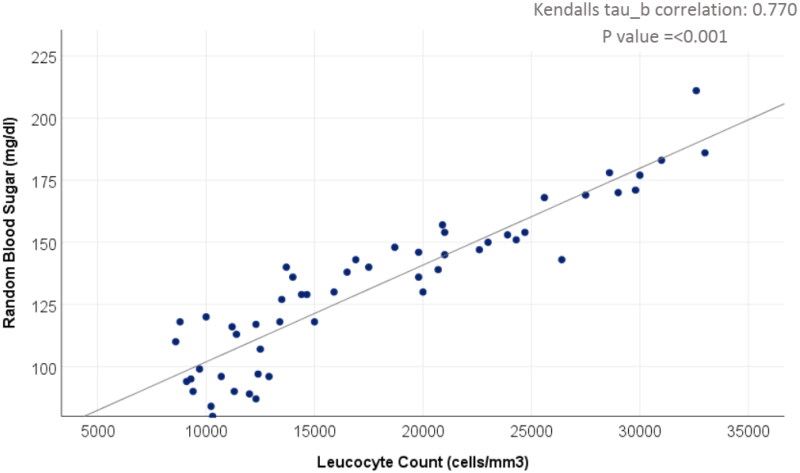
Correlation between random blood sugar and total leucocyte count.

Further correlations between the variables of interest were studied, and a significant strong direct correlation was found between RBS and TLC (*r* = 0.770, *p*-value: <0.001) (Figure 3).

**Figure 4.**
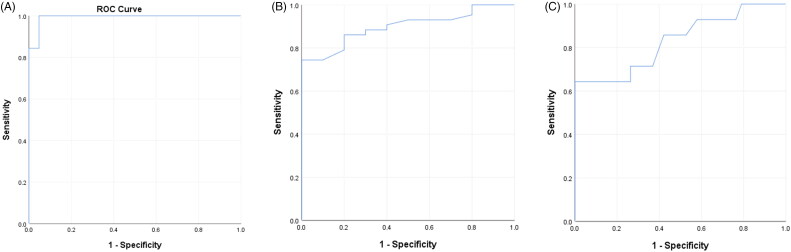
**(A)** Receiver operator characteristic curve (ROC) for total leukocyte count in detecting stress hyperglycaemia. **(B)** Receiver operator characteristic curve (ROC) for Random blood sugar in detecting leukocytosis. **(C)** Receiver operator characteristic curve (ROC) for Random blood sugar predicting complicated appendicitis.

The TLC cutoff value for predicting stress hyperglycaemia was 13,595 cells/mm^3^, as generated by the ROC curve analysis, at which the following parameters were observed; the sensitivity was 96.9%, specificity was 95.2%, positive predictive value (PPV) was 97%, and negative predictive value (NPV) was 95%. The AUC was 0.993, as represented by the ROC curve (Figure 4(A)). The cutoff value of RBS for predicting leukocytosis was 111.5 mg/dl, at which the following parameters were observed; the sensitivity was 86%, specificity was 80%, PPV was 94.9%, and NPV was 57.1%. The AUC was 0.898, as represented by the ROC curve (Figure 4(B)). The cutoff value of RBS for predicting complicated appendicitis was 141.5 mg/dl, at which the following parameters were observed; the sensitivity was 71.4%, specificity was 73.7%, PPV was 50%, and NPV was 87.5%. The AUC was 0.825, as represented by the ROC curve (Figure 4(C)).

By comparing the RBS level among different diagnoses, it showed a significant difference (p-value: 0.001). Pairwise comparisons with Bonferroni adjustment were performed to detect the different categories. It was found that the median RBS level was significantly higher in complicated appendicitis (169.5 mg/dl) compared to uncomplicated appendicitis (118.0 mg/dl) (p-value: 0.002). Also, it was significantly higher in complicated appendicitis (169.5 mg/dl) compared to intussusception (127.0 mg/dl) (*p*-value: 0.017) (Table 2).

**Figure 5.**
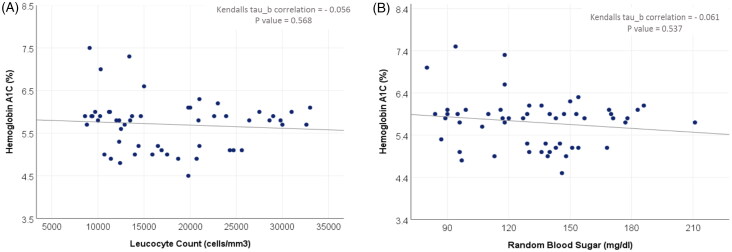
(A) Correlation between haemoglobin A1C and total leucocyte count. (B) Correlation between haemoglobin A1C and random blood sugar.

The median TLC count did not show a significant difference (16.7 × 10^3^ vs. 14.65 × 10^3^) when compared between patients with normal HbA1c and those with high HbA1c (*p*-value: 0.8). As well, there was no significant correlation between HbA1c and TLC individual values (Figure 5). However, a significantly higher proportion of patients with complicated appendicitis had a high HbA1c e.g.7 (92.9%), when compared to those with uncomplicated appendicitis (54.8%) (p-value: 0.03) (Table 3) (Figure 5(A)). HbA1c was high in 71.4% of intussusception cases.

**Table 3.** Association between haemoglobin A1C and total leucocyte count, random blood sugar and diagnosis.

	Haemoglobin A1C		*P* value
	Normal	High	
Total leucocyte count (cell/mm^3^)			
Median (range)	16,700 (10,700–25,600)	14,650 (8600–33,000)	0.800
Random blood sugar (mg/dl)			
Median (range)	138 (87-168)	192 (80 − 211)	0.878
Diagnosis *n* (%)			
Uncomplicated Appendicitis	14 (45.2)	17 (54.8)	0.030
Complicated Appendicitis	1 (7.1)	13 (92.9)	
Intussusception	2 (28.6)	5 (71.4)	

* Irreducible hernia could not be included in this analysis because it has only one case.

As the enrolled children were not diabetics, no significant correlation was found between HbA1c and RBS, which highlights that the higher levels of both were due to a non-diabetic cause, i.e. is the inflammatory state of the enrolled children with the acute surgical abdomen (Figure 5(B)).

## 4. Discussion

### 4.1. Analysis of the findings

Like most infectious diseases, a male predominance was noted in our study. Our report has yielded that acute appendicitis was the most common cause of acute surgical abdomen. Descriptive results include a higher prevalence of stress hyperglycaemia (RBS), abnormal HBA1c and elevated TLC. Analytical results displayed a linear correlation between RBS and TLC, absence of linear correlation between HbA1c and either TLC or RBS, and a significant association between abnormal high HbA1c and complicated appendicitis, in comparison to uncomplicated appendicitis. It appears that the glycaemic dysregulation is related to inflammation and its degree; as RBS was significantly higher in complicated than uncomplicated appendicitis, and intussusception.

### 4.2. Interpretation and comparison of the findings

This study was carried out on admitted children with a surgical acute abdomen to outline the role of glucose indices such as HbA1c and RBS as markers for this condition in children. These markers could be predictive markers for surgical acute abdomen, more than being definitively diagnostic ones, as they could be elevated in cases with medical acute abdomen as well. They are rather good negative tests, as compared to leukocytosis which is a good positive test. The hallmark of our study is that RBS is predictive of surgical acute abdominal conditions in children, and that HbA1c is high in most of those cases and differs significantly between complicated and uncomplicated appendicitis.

Appendicitis was the most common primary diagnosis. This finding agrees with meta-analyses, randomized controlled trials and systematic reviews relating to appendicitis, as done by Humes & Simpson (2006) [13].

Regarding the total leucocytic count can as an inflammatory marker, a former study has concluded that measuring TLC substantially increases the detection rate and predictivity for of bacterial infections [14]. Additionally, TLC may be of more value in detecting the inflammatory state than than either C-Reactive Protein (CRP) and/or Erythrocyte Sedimentation Rate (ESR). This was detected in a prior report in which it was found that TLC, ESR and CRP in children with acute appendicitis are significantly higher as compared to those who have nonspecific abdominal pain [15]. Furthermore, the predictive value of TLC in diagnosing acute appendicitis was investigated, and found to have a with sensitivity and specificity of 51.9% and 89.5% respectively. Besides. It showed higher discriminatory values between complicated and uncomplicated appendicitis, *p* < 0.001 [16]. Obviously, CRP may exhibit a delay to rise of up to12 h after the onset of symptoms of appendicitis in children, andmay have a delayed peak of up to 48 h, thus lags behind TLC [17], consequently, it may be less reliable at the time of referral. Therefore, it was not included in our study [18].

Higher TLC was associated with complicated appendicitis in the enrolled children, in our study. ***Feng*** et al. ***(2020),*** similarly found that the TLC, age and symptomatic duration combinedly predict complicated appendicitis in children younger than five years old [19]. Our study found another association, as well, that could be used as a prediction for complicated acute abdomen in general and particularly complicated appendicitis, which is the random blood sugar in children without diabetes mellites. Higher values of random blood sugars were linked to the presence of complicated appendicitis, which is intuitive in the setting of stress hyperglycaemia. ***Davis*** et al. ***(2017)*** detected a parallel result that higher levels of stress hyperglycaemia are linked to higher rates of perioperative complications and hospital mortalities in non-diabetic surgical patients. The term stress hyperglycaemia signifies transient elevations in blood glucose in non-diabetic patients during acute illness or stress [20]. Detection of children with stress hyperglycaemia is recommended in children with acute abdomen, as previous randomized controlled studies have revealed that its treatmentdiminishes complications and improves overall outcomes. In general surgery, the development of perioperative hyperglycaemia is linked to at least a 4-fold increase in complications and a 2-fold increase in death compared to patients maintaining normoglycaemia [21–26]. We did not link our findings to outcomes, but this could be one of our future directions. 

We investigated the link between the random blood sugar and TLC and found a direct significant correlation between those two variables, and an association between both parameters on the one hand, and the severity of inflammation pertinent to the acute abdomen on the other hand. ***Xu*** et al. ***(2013)*** observed a similar close association, that elevated leukocytic counts are predictive of higher levels of hyperglycaemic emergencies. Hence, leukocyte counts could add valuable information to reveal the presence of hyperglycaemic crises and acute infection [27]. ***You*** et al. ***(2019)*** found that high TLC combined with high blood glucose levels at admission were independently linked to in-hospital mortality [28]. In that study (***You*** et al. ***(2019)***), it was concluded that the combination of TLC and blood glucose level appeared to be better predictors than either TLC or blood glucose alone.

In the current work, we did not study blood sugar only, as a biomarker for acute surgical abdomen in children, but also attempted to investigate HbA1c, as well, as an inflammatory marker. An earlier study detected that the haemoglobin glycation index (HGI), which is based on HBA1c, could be a marker of risk associated with inflammation, independent of blood sugar measures [6]. A previous positive correlation between HBA1c and other inflammatory markers, such as ferritin, was found in a preceding research [29]. Notably, HBA1c is suggested to be related to macrovascular complications in non-diabetic patients and their inflammatory state [30,31].

A statistically rigorous association between HBA1c and TLC was not found in our study. This finding disagrees with the finding of ***Hong*** et al. ***(2018)*** who detected that HbA1c levels were positively associated with TLC levels. In the latter study, adjusted HbA1c levels were increasing across the TLC quartiles even within their normal range (*p* < 0.001), after adjusting for the confounders, such as age, gender, fasting plasma glucose, education, smoking , anthropometry, hypertension, lipid profile, and anaemia. However, this disagreement may be due to the larger sample size, and recruitment of some diabetic patients in the latter study, unlike ours. Nevertheless, and even more importantly, 92.9% of our patients with complicated appendicitis exhibited a high HbA1c, compared to 54.8% of our patients with uncomplicated appendicitis, which is consistent with several studies which have shown that the increased inflammatory responses influence HbA1c levels [6,32,33]. Even in nondiabetic subjects, HbA1c was found to be positively associated with inflammation, as a part of the metabolic response to surgical pathology [34–36].

### 4.3. Future directions

Further research is needed to study the underlying mechanisms and address their prognostic value. The underlying biochemical mechanisms responsible for the detected disparity between HbA1c and blood glucose are yet to be explored. In our study, no significant association between random blood sugar and HbA1c was detected. Thus, the role of HBA1c for inflammation and oxidative stress was suggested [30]. We could safely hypothesise that the elevated HbA1c in our cohort was not related to the glycaemic status of the patient.

Despite the lack of correlation between HbA1c and TLC, an abnormally high level of HbA1c was observed in a considerable number of patients in our study. This allows us to propose the role of the HbA1c as an inflammatory marker in the setting of normoglycaemia. Therefore, either the random blood sugar or HbA1c could be affected, thus they are better to be used synergistically, as biomarkers for acute surgical abdomen, than if each is used alone.

## 5. Conclusion

The current study has identified that random blood sugar and HbA1c are relevant independent markers for inflammation in children with surgical abdomen. Therefore, surgical pathologies as causes for stress hyperglycaemia and glycaemic dysregulation should be strongly considered.

## Recommendations

Preoperative surgical hyperglycaemia is linked to postoperative undesirable outcomes. Hence, the identification of children with preoperative stress hyperglycaemia is recommended. This is a form of risk stratification for children with acute surgical abdomen, which may guide the subsequent management plans.

## Study limitations

Other inflammatory markers, of potential interest, such as Ferritin and Erythrocyte Sedimentation Rate (ESR) could not be done, as the study was done in the emergency setting where those laboratory tests were not available. Additionally, the strict inclusion criteria resulted in a relatively small sample size.

## Data Availability

The data is available with the corresponding authors upon a reasonable request.

## Abbreviations

HbA1c: Glycated Haemoglobin
RBS: Random Blood Sugar
TLC: Total Leukocytic Count
DM: Diabetes Mellitus
ROC: Receiver Operator/Operating Characteristic Curve
AUC: Area Under the Curve (in ROC)
CRP: C-Reactive Protein
ESR: Erythrocyte Sedimentation Rate
